# Ego network analysis of the trophic structure of an island land bird through 300 years of climate change and invaders

**DOI:** 10.1002/ece3.8916

**Published:** 2022-05-20

**Authors:** Jens M. Olesen

**Affiliations:** ^1^ Department of Biology Aarhus University Aarhus C Denmark

## Abstract

Ego net analysis is a well‐known practice in social sciences, where an ego net (EN) consists of a focal node, the ego, and its links to other nodes, called alters, and alter–alter links may also be included. An EN describes how a focal node is embedded in its interaction context. Here, I introduce EN analysis to ecology in a study of the trophic network of a sub‐Antarctic land bird, Lesser Sheathbill (*Chionis minor*). Data originate from the sheathbill population on Marion Island in the Southern Ocean. The bird is ego and its enemies and food are alters. The EN is organized along three dimensions: habitat, interaction type, and time (from before human arrival in 1803 and until a future year 2100). Ten EN descriptors are defined, estimated, and used to track the 300 years of change in sheathbill EN structure. Since 1803, the EN has passed two major, but reversible shifts—seal exploitation in the 19th century and presence of cats from 1949 to 1991. These shifts can be read as structural changes in the sheathbill EN. In the future, a third, perhaps irreversible change is predicted, driven by climate change and a surprising, recent shift to seabird predation by House Mouse, the most detrimental of all extant invaders on Marion. In a warmer and drier future, the mouse will proliferate, and if this forces seabirds to abandon the island, their accumulation of detritus runs dry, starving a rich invertebrate detritivore fauna, which also is a key food source to sheathbills. These detritivores together with plants have also constituted the main food sources of mice. The EN descriptors quantify that story. In the future, these events may lead to a collapse of the island ecosystem, including extinction of the sheathbill—unless plans for mouse eradication are implemented.

## Graphical abstract

Ego net analysis is introduced as a new tool to ecology, describing the network context of a single bird species and its interacting species. During the last 200 years, the ego net of this bird has passed two major disturbances, seal hunting and cat introduction. In the present century, a third disturbance is expected, driven by climate change and invaders. This is visualized by ego net analysis.

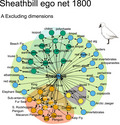

## 1 INTRODUCTION

Here you will find the proverb befitting, Sancho said, tell me, with whom you are together, [and] I’ll tell you, who you are … Ingenioso Cavallero Don Qvixote de la Mancha Miguel de Cervantes Saavedra, 1615

Network analysis has become famous for its ability to deepen our comprehension of the complexity of hundreds or even thousands of data points and their inter‐connections (e.g., Estrada, 2011; Delmas et al., 2019). However, it may also, more modestly and perhaps counter‐intuitively, recede focus by turning toward topological qualities of a single focal node of key interest. This is a well‐known practice in social sciences (Perry et al., 2018; Vacca, 2018)—for example, a study of a focal person and her friends can be accomplished without any further knowledge about the complete network of all persons and their friends. An ecological analogue could be a focal species and its interacting prey, predators, pathogens, etc. Such an approach would bring us back in the middle of the road between “old‐school” autecology and modern network ecology, and it introduces a kind of network in its own right, but also a network, that is a building block of larger and more complex multispecies networks.

**FIGURE 1:**
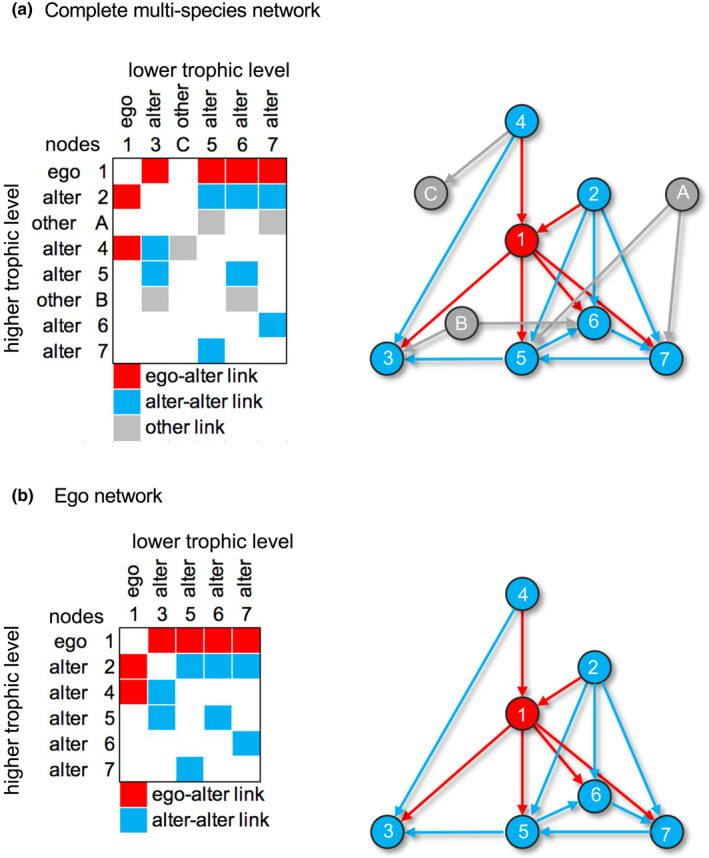
Toy model of a complete network and one of its ego nets. (a) **complete species network** shown both as an 8 × 6 species incidence matrix and as a node‐n’‐link model: red cells for ego and ego‐alter links, blue for alters and alter‐alter links, and gray for other nodes and links. Rows and columns are higher and lower trophic level species, respectively. However, some species are present at both higher and lower level, for example, alter 5 consumes alters 3 and 6, but is consumed by ego, alters 2 and 7, and “other A.” (b) **ego net** of species 1 (“ego”). The ego consumes alters 3, 5, 6, and 7, but is consumed by alters 2 and 4. The net also includes eight alter‐alter links (blue) between the six alters. The nodes “other A‐C” in (a) are not included in (b), because they miss direct links to ego, and thus, by definition, are outside the ego net

In social network theory, such single‐focal node networks are called *ego networks* or just *ego nets* (e.g., Crossley et al., 2015), and by playing scientific broker, I here introduce the concept to ecology. In an ego net, the key species is the *ego*, and its linked, associated species are the others, the *alters*. Thus, an ego species is the central node in its own star‐shaped network. Links between ego and alters are called *ego–alter links*, and if data are at hand, an ego net also includes *alter–alter links*. Thus, a complete multispecies network with *n* interacting species may be deconstructed into *n* ego nets (Figure 1).

Albeit analyses of complete networks and ego nets are related, they differ in research foci. The former is a model of natural complexity in general, revealing emergent properties. The latter, on the other hand, is a concrete object, even a kind of “trait,” visualizing the contextual biotic properties of an ego species. Thus, if the question is about individual species and their behavior, then ego net is the way to go, whereas if the question is about link patterns, diffusion of effects in an assembly of species, etc., one would use a complete network approach. However, both may be combined into broader analyses. As a first step, the complex network analysis may discover single species of key importance to topology, ecology, or evolution of the complete network. These species may then, in a second step, get their ego net analyzed. However, research may also run in the opposite direction. In a preliminary complete network analysis or in a complete network analysis of a very large network, where one most likely misses data, a first step could be to analyze the ego nets of a small sample of species, followed by predictions about total structure of the complete network. Most likely, however, our interest is not complete networks at all, but more a deeper understanding of individual species. Here, one would go directly for the ego net, because a detour around the complete network would be irrelevant.

Besides size of an ego net, it is informative to get a picture of its heterogeneity and one aspect of this is modularity, that is, expression of modules. In general, modules are densely linked groups of nodes, and most networks—social, biological, and engineering—show a strong modular signature. Thus, whatever network, its basic components, at a scale larger than individual nodes and links, become modules (Fortunato, 2010; Guimerà & Amaral, 2005; Newman, 2006; Olesen et al., 2007; Sales‐Pardo, 2017). Some go way further and argue that modules constitute the critical level in the organization of life (Hartwell et al., 1999). Thus, a network may be defined as a structure of 1‐several inter‐connected modules delimited by researcher‐defined boundary specifications. Such a network definition places the module in the center of our ultimate quest for understanding natural complexity. Since we routinely operate with the term “species niche” and cautiously navigate around its murkier corners (Schoener, 1990), then perhaps we should also talk about “module niche,” because species and links in a module and their natural history constitute the ecological content of the module, and their collective functionality, the module niche, becomes an emergent property (e.g., Jeong et al., 2000). Generally, modularity is a feature, which adds stability to a network through damage embankment (Pimm, 2002; “the watchmaker parable” in Simon, 1962).

In particular, an ego‐net analysis is a strong tool in comparisons of different entities, times, or localities. If we get tens or perhaps even hundreds of ego nets, we may compare the ways in which species influence and are influenced by their local biotic environment (for social science analogues, see Marsden, 1987).

Here, I (i) introduce ego‐net analysis to ecology, (ii) use this analysis to describe the trophic ego net structure of an island land bird species, and (iii) track the dynamics of this net through a time span of 300 years—from prior to human arrival onto the island in 1803 and until a future year 2100—in order to locate and describe major temporal shifts in network structure.

The bird of choice is the Lesser Sheathbill (*Chionis minor*), which lives on the sub‐Antarctic Marion Island (Burger, 1982)—a supposedly simple ecosystem. Using this example as a starting point, I want to understand, how a few “hardcore” oceanic island land birds still survive in a world, that has witnessed the extinction of thousands of island birds (Steadman, 1995), and as my tool I use ego net analysis.

## 2 MATERIALS AND METHODS

### 2.1 Marion Island

Marion Island (47°S, 38°E) and its neighbor Prince Edward Island constitute a protected South African archipelago in the Southern Ocean. The island has an extent of 290 km^2^, and its highest peak reaches 1230–1280 m above sea level. With its latitudinal position in the “roaring forties” and close to the “furious fifties” (McCann, 2018), Marion is a typical sub‐Antarctic island in climate, geology, and ecology (Chown & Froneman, 2008). Its land ecosystem is driven by sea‐land commuting animals, that is, Marion is also a classic seabird island (*sensu* Anderson & Mulder, 2011). My study exemplifies, how many, perhaps most islands devoid of alien seabird predators in their ecology is driven by a sea‐land pipeline, importing marine organic matter (Croll et al., 2005) and, more generally, that islands, because of their seabird colonies and human traffic (McCarthy et al., 2022), cannot be seen as ecological isolates, albeit they may be so evolutionarily (Borregaard et al., 2017; Holt, 2010).

### 2.2 Ego

Lesser Sheathbill (*Chionis minor*) is a 600 g land bird with black beak and white plumage. It is distributed as four subspecies on sub‐Antarctic islands. I focus upon the population on Marion (subspecies *marionensis*). Here, the sheathbill has a central position as a sedentary generalist (Burger, 1980; Burger & Kirwan, 2020; McClelland, 2013). It forages both on land, in tidal zone, and in near‐shore waters. During summer, it associates with colonies of seabirds and mammals, consuming carrion and food scraps and stealing eggs and chicks. As side dish, the sheathbill takes invertebrates, especially detritus‐ and plant‐feeding weevils and moths (Burger, 1981). In winter, when most seabirds have left, it moves more out into the tidal zone, eating invertebrates and algae (Jouventin et al., 1996) or further inland (Burger, 1981; Huyser et al., 2000; Verheyden & Jouventin, 1991). The sheathbill also benefits from food leftovers from overwintering King Penguin (*Aptenodytes patagonicus*) (Burger, 1984). Its bare parts around eyes and beak may be related to its vulture‐like feeding habits, for example, when it hollows out seal carrion. It nests in cavities and cracks in rocky areas (Burger, 1980).

The sheathbill is of great interest to conservation and island biology, because it still survives! A fact that runs counter to the general history of oceanic island birds. Soon after humans and their animal companions settled on oceanic islands, waves of extinction took place, particularly among land birds—one reason being their lack of previous exposure to mammal predators and pathogens (e.g., Biber, 2002; Blackburn et al., 2004). During the past 400 years, more than 90% of all bird extinctions took place on islands (e.g., Johnson & Stattersfield, 1990). Although, we know why most of these island birds went extinct, it is of general value to conservation and island biology to improve our understanding of the ecology of those few, such as the Lesser Sheathbill, that withstood the calamities and survived. These are my arguments for a formal analysis of this particular island species.

### 2.3 Data

Unfortunately, I have never visited Marion, but many others have and consequently, it is very well researched (e.g., Chown & Froneman, 2008), I repeat—very well. Since its discovery, a steady flow of commercial and scientific expeditions has paid visits to Marion and gathered detailed information about its biota (e.g., Cooper, 2008), but alongside also mediated an influx of invaders. The general invasion biology and arrival time of these invaders are discussed at length in many publications (e.g., Greve et al., 2017, Supplementary Material S5). Marion may even have the best‐known natural history and biodiversity of any small oceanic island.

I perused this rich literature for any records about sheathbill's feeding interactions (Supplementary Material S2), and all these published interactions were the input of my analysis. Interactions have, like species, a habitat, in which they take place, and I recorded this as a link trait. However, I did not include “eco‐logical” interactions, if they stayed unrecorded, and marine alter–alter links were also left out, because the required data were missing. Apparent and exploitative competitive interactions may be represented by some of the alter–alter links (Holt, 2009), but to include them here would be pure guesswork. (However, for a tentative analysis of potential indirect links, see below in Appendix 1). Finally, I ignored interaction strength, because I was unable to find a common “fitness currency” for the different kinds of link and body size range; the latter spanning from bird lice to sea elephant. However, since all links were trophic, their “valence” was negative.

Seventy‐eight percent of all alters were resolved to either species or genus (Supplementary Material S1). Ten nodes were aggregates of several species, among them the marine food resources fish, squids, copepods, algae, and tidal invertebrates. Without this sea connection, the analysis would be meaningless, because of the dominant importance of seabirds and sea mammals as drivers of organic matter input to the island. Broadly speaking, if link strength is not included in an analysis, no information about the basic network structure seems to be lost by aggregating taxa (Cohen et al., 1993; Martinez, 1991).

The most serious invader on Marion is House Mouse (*Mus musculus*) (e.g., Angel et al., 2009; Simberloff, 2009), but surprisingly enough it was not part of sheathbill's ego net, because to this day any evidence of direct feeding interactions with sheathbill is lacking, but exploitatively, the mouse, for sure, affects the latter by its consumption of invertebrates (Burger & Kirwan, 2020; see later and also Appendix 1). To a minor extent, sheathbill probably also forages on carrion of mouse (McClelland, 2013). Finally, the island has no records of native biotic pollination and seed dispersal interactions, which is a generic signature of sub‐Antarctic islands (Convey et al., 2010).

Besides the cat, being present on the island from 1949 to 1991, and which predated upon sheathbill (Huyser et al., 2000), and sheathbill scavenging on human garbage at the research station, there are, to the best of my reading, no recorded direct feeding interactions between sheathbill and invaders, which makes the bird unusual, and this particular chapter of its ecology is perhaps a main key to its persistence.

In a list of the World's 100 worst invasive alien species from the *IUCN* (International Union for Conservation of Nature) (Supplementary Material S3) and a list of cold‐preadapted taxa (Supplementary Material S4) (Duffy et al., 2017) I looked for potential future invaders and new sheathbill alters. However, my analysis only predicted two new interactions, involving sheathbill (for details, see Supplementary Materials S3 and S4). These were an ego–alter link (*Chionis minor* [node 186]–*Fucellia tergina* [Seeweed Fly, node 369]) and an alter–alter link (*Fucellia tergina* [node 369]–detritus [node 336], Supplementary Materials S1 and S2, S4). Both were added to the database.

### 2.4 Ego net analysis

As stated above, ecological ego net analysis is a tool to visualize and quantify how a single species or any other entity is embedded in its net of interactions. Strongly inspired by social network studies, my analysis introduces a few, simple descriptors of ego nets (Crossley et al., 2015; Perry et al., 2018)—descriptors, that may turn out to be of value in ecology. Since ego nets often are small, most descriptor values can be calculated in a simple spreadsheet. However, much network software is also available and here, *R*‐libraries (especially *Igraph* and *bipartite*) and *Pajek* (Nooy et al., 2011) are used. I tether my analysis to ten descriptors, which together unfold the basic complexity structure of the network, but many more are in the social sciences' toolbox (Crossley et al., 2015; Perry et al., 2018; Vacca, 2018). Descriptors 1–5 measures network size and descriptors 6–10, network heterogeneity. Below, each descriptor is defined.

Description of ego net size.

**FIGURE 2:**
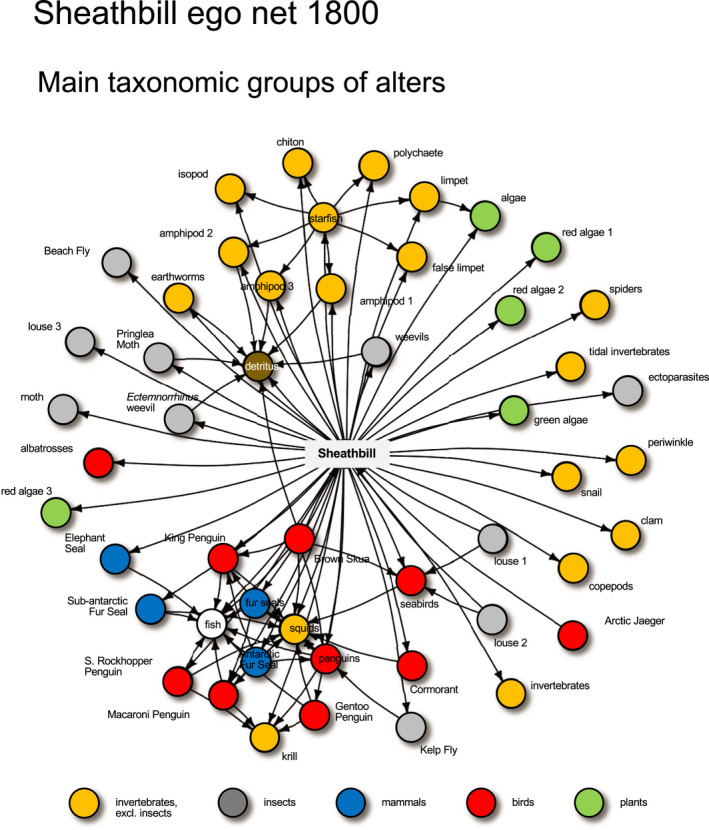
Ego net of sheathbill (*Chionis minor*) before arrival of humans in the summer of 1803/1804. The ego, the sheathbill, is in the center and arrows point from consumer to resource. The net shows taxonomic groups. Data from all habitats and interaction types are pooled, that is, dimensions are ignored (see 2.4, Descriptors 9–10). (See Supplementary Material S1 for scientific names)

**Descriptors 1–2**. *Ego net size* is given as number of alters, *N*, and links, *E*. *N* is also termed degree centrality of ego. *N* and *E* are the most basic network descriptors, but perhaps still the most informative, because they scale with other network and node properties, for example, body size (West, 2017).
**Descriptors 3–4**. *Ego net link density* is a measure of connectedness (connectance or standardized link number per node) among nodes. Intuitively, link density is a simple measure, but it can be defined in several subtle ways, that is, on different levels of realism, and one should be careful about how it is defined. Here, I introduce two densities, *d*_1_ and *d*_2_. Total link density *d*_1_—The ego net is seen as 1‐modal, that is, all nodes (*N* + 1, ego included) may potentially interact and all links *E* are directed. The only constraint is the exclusion of potential intra‐node links or loops, that is, cannibalism. Thus, *d*_1_ = 100 · *E*/[*N* (*N* + 1)]. Alter link density *d*_2_—The ego net is seen as 2‐modal, that is, all alter nodes (*N*, ego excluded) belong to one of two kinds and only links (*E*–*N*, *N* is number of links to ego) between kinds are considered. Here, the two kinds are consumers and their resources and links are directed. By sorting alters into either consumers or resources, I exclude trophically forbidden (unrealistic) links, for example, the amphipod *Hyale grandicornis* (#298, Supplementary Material S1) consuming the starfish *Anasterias rupicola* (#300) (Izquierdo‐Palma et al., 2021; Olesen et al., 2011; however, see the issue of structural holes in Descriptor 5). Thus, *d*_2_ = 100 · (*E – N)*/(*N*_consumers_ · *N*_resources_ – *N*_consumer = resource_). *N*_consumers_ and *N*_resources_ are number of consumer and resource nodes, respectively. A few nodes are both consumer and resource, and their potential loops are excluded (*N*_consumer = resource_). The network in Figure 1b, for example, has seven nodes (ego + six alters) and 14 links, and *d*_1_ = 100 · 14/[6 · (6 + 1)] = 33, and *d*_2_ = 100 · (14 – 6)/(5 · 4 – 3) = 47. In my calculations, I just use a spreadsheet, but *Pajek* also calculates densities (Nooy et al., 2011: 369ff for 1‐mode input format and its definition of densities): Choose *Info*>Network>*General*.
**Descriptor 5.** *Effective size of ego net*. Presence of a link tells a piece of natural history, but so does its absence. The latter is called a *structural hole* (Burt, 1992; Nooy et al., 2011; however, see the issue of forbidden links in Descriptors 3–4). A measure of number of structural holes in an ego net is *effective network size* $N_{\text{eff}}=N‐\sum d_{j}/N$, where *d*_j_ is number of alter links for alter *j* (Perry et al., 2018). Normalized effective size is called *efficiency* = *N*_eff_/*N*. Put simply, effective size is equal to actual size minus redundancy.
  Description of ego net heterogeneity.
**Descriptor 6**. *Ego net betweenness*. By definition, ego is the most central node in the network; even if an alter, at least in theory, might achieve a similar linkage level, *N*, as ego. To measure how strong the central position of ego is, several centrality descriptors are available, for example, *N* and betweenness *b*_ego_ (Martín et al., 2010). The latter is number of all shortest paths between all alter pairs, that needs to pass ego, ignoring link direction. This number is normalized by dividing with the maximum value of *b*, that is, when there are no alter–alter links. In general, path length between two nodes is measured as number of separating links. On Marion, for example, Pringlea Moth (*Pringleophaga marioni*) and starfish are connected by two paths: the shortest one being Pringlea Moth—sheathbill—starfish, and a longer, 3‐link path being Pringlea Moth—detritus—amphipod 3 (*Jassa falcata*)—starfish, which does not pass the ego (Figure 2). If an alter pair has two shortest paths, and only one of them passes ego, then it counts as 1/2 in the calculation of *b*. To get *b*, choose *R*>*Igraph*>*centr_betw* or *Pajek*>Net>*Vector*>Centrality>*Betweenness* (both give the same result, but third decimal may vary). In general, betweenness is a “global” (here synonymous to “ego”) network property, that is, based on information about the entire net, whereas degree centrality, *N*, is a “local” network property, that is, based on information about the 1‐link neighborhood of a single node.
**Descriptors 7–8**. *Ego net heterogeneity (module ratio*, *modularity*, and *fragmentation index)*. These descriptors focus upon modules of alters. Level of modularity can be expressed as a *module ratio*, *M*_ratio_ = (*N*_m_ – 1)/(*N* – 1), where *M*_ratio_ is a normalized measure of number of modules, and *N*_m_ is number of modules. In social sciences, *M*_ratio_ is called component ratio (Perry et al., 2018). I use the algorithm *Generalized Louvain* (*R*>*Igraph*>*cluster_louvain*) to detect modules both without and with data about dimensions (see Descriptors 9–10 for more about dimensions; see *R*—input files etc. in Supplementary Material S5). Generalized Louvain seeks a node partition, that gives a maximum modularity level *Q*, that is, maximum ratio between link density within and between modules. It optimizes *Q* in a “greedy” manner, that is, fast and heuristically (for more details, see for example, Blondel et al., 2008). Other algorithms are also available, but may give different modularity resolution, because module boundaries are relative changes in link density and these changes are detected slightly differently by algorithms. Only *M*_ratio_, but not *Q*, is included here. Unfortunately, *M*_ratio_ is insensitive to variation in module size in the net, which certainly affects flow of information. This problem can be addressed by using the *fragmentation index* $F=1‐\sum r_{\mathrm{ij}}/\left[N\left(N‐1\right)\right]$, which is a normalized measure of module size variation. It gives proportion of alters, that cannot link directly or indirectly to each other along a path in the ego net, excluding any path passing ego. In order to calculate *F*, a reachability matrix is constructed with cell elements *r*_ij_ = 0 or 1. “0,” if two alters *i* and *j* only are connected through ego and “1,” if it is possible to get from *i* to *j*, without passing ego.
**Descriptors 9–10**. *Ego net diversity (dimensions and layers)*. Alters vary, for example, in their taxonomy, habitat, and interaction type (trophic role). Each of these is a network dimension and the contribution of each dimension to network diversity can be quantified. Strictly speaking, there is no such thing as a diverse ego net; one has to specify, which dimension is on the agenda.

Along each of the two dimensions habitat and interaction type, I focus upon distinct points or here layers, about which empirical data are available (Eklöf et al., 2013). As diversity index, plenty are at hand; social scientists often use *Blau's heterogeneity index* $H=1‐\sum p_{k}^{2}$, where *p*_k_ is the proportion of ego's alters, that belongs to a given layer, and its standardization, the *Index of Qualitative Variation* IQV=H/(1‐1/n), where *n* is number of alter layers (Blau, 1977). *H* is familiar to ecologists as Simpson's diversity index. *p*_k_ may, for example, be proportion of alters, that interacts with sheathbill in the tidal zone along the habitat dimension.

Descriptor 9 (*H*_h_, alter diversity among habitat layers): Habitat dimension is resolved into an ordinal series of three layers, involving sheathbill, *viz*. land, tidal zone/seashore, and sea.

Descriptor 10 (*H*_i_, alter diversity among interaction type layers): Interaction type dimension has a nominal set of four layers, involving sheathbill, *viz*. detritivory, herbivory, predation, and parasitism.

I did not calculate alter diversity along the temporal dimension, but time is represented by an interval series of seven or eight layers (time snapshots, time‐slices, “years”), *viz*. 1800 (prehuman), 1820, 1930, 1975, 1995, 2020 (today), and 2100 with or without invading mouse (for a detailed description of each “year,” see below in Appendix 2). The temporal dimension is analyzed in section 3.2.

The concept of layer is used here, because it, by now, is well established in studies of multilayer networks (Costa et al., 2020; Hervías‐Parejo et al., 2020; Hutchinson et al., 2019; Pilosof et al., 2017). However, it is a discrete entity, and as such does not, in my opinion, give a fully satisfactory description of nature, for example, none of the dimensions in this study are truly categorical.

Obviously, more dimensions would add to a description of ego net diversity, for example, season and trophic level. Season would highlight that the sheathbill retracts its foraging from the marine predation layer during winter, when seabirds also leave for their winter‐foraging grounds. Along the trophic level dimension, sheathbill would be scored as a detritivore, predator, prey, herbivore, and host to parasites. The sheathbill is, for example, a prey to Brown Skua (*Catharacta antarctica*) and Arctic Jaeger (*Stercorarius parasiticus*) and a host to parasitizing chewing lice (Philopteridae, louse species 1 and 2 in Figure 2).

Within a given dimension, other descriptors are layer relevance, connectivity, and correlation, which estimate topological consequences of removing or adding particular layers, measured in the currencies “species,” “links,” or “modules” (for details, see Berlingerio et al., 2011). Finally, it is important to keep in mind, that dimensions also may be correlated, for example, sea is dominated by predation and land by detritivory and herbivory.

Standing alone, these descriptors do not make much sense, because measurement is not understanding; they are developed for comparative purposes. I make that step here, tracking the temporal dynamics of the descriptors over the course of 300 years (for details about temporal analysis of ego net in general, see Crossley et al., 2015: chapter 7). I do this temporal study, because I want to know, if two well‐described, major ecological disasters on Marion, *viz*. seal exploitation and presence of cats, are detected by the descriptors and to what extent these descriptors further predict any future collapse or regime shift. Finally, as usually assumed in temporal network analysis, a recorded link, once observed, stays in the network as long as its interacting species pair stays.

## 3 RESULTS

### 3.1 Description of the sheathbill ego net for year 1800, that is, before arrival of humans

The ego net of sheathbill in the prehuman time layer, before any arrival of whalers and sealers in the austral summer of 1803/1804, is my baseline (Figure 2). The sheathbill interacted with several groups of plants and animals and consequently had a wide trophic niche. Below, I calculate its descriptor values.

**FIGURE 3:**
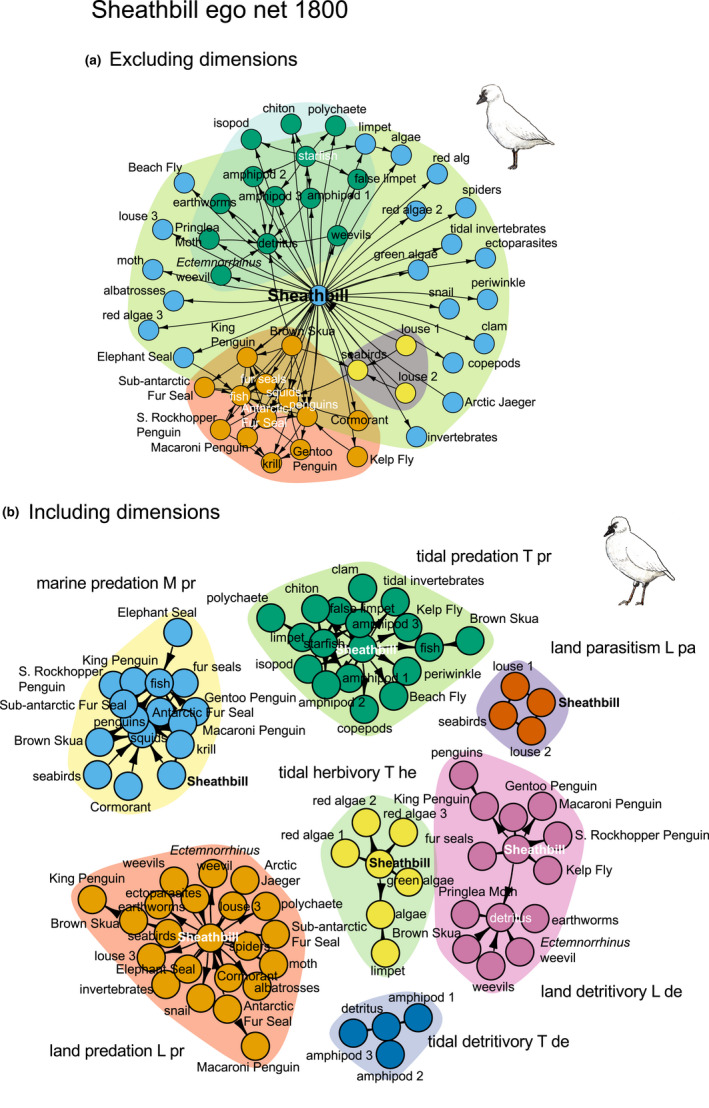
Ego net of sheathbill (*Chionis minor*) before arrival of humans in the summer of 1803/1804. (a) habitat and interaction‐type dimension data are excluded., that is, each taxon is only represented by one node. Modules are distinguished by color. (b) habitat and interaction‐type dimension data are included, that is, the same taxon may be represented by several nodes, for example, skua‐marine‐predation and skua‐land‐detritivory. Modules are distinguished by color. (See Supplementary Material S1 for scientific names)

**Descriptors 1–2**. *Ego net size*. Size of the sheathbill ego net was *N* = 50 alters (49 alter taxa and the alter node “detritus”) and *E* = 104 links, that is, 50 ego–alter links and 54 alter–alter links (Table 1, Supplementary Material S1 and S2).
**Descriptors 3–4**. *Ego net link density*. Total link density was *d*_1_ = 100 · *E*/[*N* · (*N* + 1)] = 100 · 104/[50 · (50 + 1)] = 4.1%, and alter link density was *d*_2_ = 100 · (*E – N*)/(*N*_consumers_ · *N*_resources_ – *N*_consumer = resource_) = 100 · (104–50)/(25 · 46–21) = 4.8% (Table 1).
**Descriptor 5**. *Effective size of ego net*. Effective size of ego net was *N*_eff_ = 50 – (104–50)/50 = 48.9. This means, that sheathbill was connected to 48.9 “effective alters” or independent, nonredundant sources of influence. Efficiency = 48.9/50 = 0.98 (Table 1).
**Descriptor 6.** *Ego net betweenness*. Level of ego betweenness was *b*_sheathbill_ = 0.90, that is, 90% of all shortest paths between alters had to pass ego (Table 1).
**Descriptors 7–8**. *Ego net modularity (module ratio*, *modularity*, and *fragmentation index)*. First, I ran a modularity analysis without information about dimensions (Figure 3a). Modules did not overlap, except for the ego, which was a member of all modules. The net had two large modules, including 14 (brown‐colored) and 13 (green‐colored) alters, and a small module of three alters (yellow‐colored). The remaining (50 – (14 + 13 + 3)) = 20 alters were only connected to ego (light blue‐colored nodes—two of these nodes, however, also had a link to the large modules), that is, each of them, together with ego, made up their own module. Thus, the net got a total of *N*_m_ = 23 modules: one 15‐node “brown” module (14 alters + ego), one 14‐node “green” module (13 alters + ego), one 4‐node “yellow” module (3 alters + ego), and twenty 2‐node “light blue” modules (one alter + ego). Thus, *M*_ratio_ = (23 – 1)/(50 – 1) = 0.45 (Table 1). *Fragmentation index F* = 1 – [33 · (33 – 1)] /[50 · (50 – 1)] = 0.57. Thirty‐three alters could reach each other without passing ego (Figure 3a, Table 1). Second, I added information about the two dimensions habitat and interaction type and by doing so, the network got 84 alters (Supplementary Material S5). Number of alter nodes was now higher, because 52% of all nodes were present in several layers in each dimension. King Penguin, for example, had three node representations: (1) as detritus source on land, because sheathbill eats penguin remains (the detritivory layer in the interaction type dimension and the land layer in the habitat dimension), (2) as prey on land, because skua eats penguin (the predation layer in the interaction type dimension and the land layer in the habitat dimension), and (3) as predator of fish at sea, because penguins eat fish, and also as prey at sea, because fur seals eat penguin (the predation layer in the interaction type dimension and the sea layer in the habitat dimension). Now, the ego net consisted of seven nonoverlapping modules (Figure 3b): three modules on land: (1) L pr (land predation), (2) L de (land detritivory), and (3) L pa (land parasitism); three modules in the tidal zone: (4) T de (tidal detritivory), (5) T he (tidal herbivory), and (6) T pr (tidal predation); and one at sea: (7) M pr (marine predation). The three predation modules (L pr, T pr, M pr) had a nested, submodular structure. At present, a tool is lacking to express *M*_ratio_ and *F* for networks with a nested, submodular structure.
**Descriptors 9–10**. *Ego net diversity (dimensions and layers)*. Along the habitat dimension, sheathbill interacted with 29 of its 50 alters on land, 23 in tidal zone, and 15 at sea, that is, 16 of the 50 alters were present in more than one habitat (15 alters lived in two habitats and Brown Skua in all three) (Supplementary Material 1). Thirty‐nine, 32, and 33 links were observed on land, in tidal zone, and at sea, respectively (Supplementary Material S2). Although possible, no link was reported in more than one layer of a dimension. Diversity of alters across habitat layers was *H*_h_ = 0.66 and *IQV* = 0.996 (Table 1). Along the interaction type dimension, sheathbill was active in four interaction type layers, interacting with 16 alters by detritivory links, six by herbivory links, four by parasitism links, and 78 by predation links. Seventeen of the 50 alters were involved in more than one interaction type (never more than two, *e*.*g*., the Kerguelen Earthworm (*Microscolex kerguelarum*) was both a decomposer and a sheathbill prey). Diversity of alters across interaction type layers was *H*_i_ = 0.41 and *IQV* = 0.545 (Table 1). Thus, the habitat dimension added more diversity to the ego net than the interaction type dimension (*IQV* = 0.996 *vs*. 0.545), because the latter was dominated by one layer, *viz*. predation (75% of all links).

**TABLE 1:** 300‐years of dynamics of ego net descriptors for Lesser Sheathbill. Two year–2100 scenarios: without (no) and with (+) mouse

Descriptors	1800	1820	1930	1975	1995	2020	2100 no mouse	2100 +mouse
1 Number of alters *N*	50	51	46	52	47	46	50	42
2 Number of links *E*	104	112	86	107	98	96	102	88
3 Total density *d*_1_	4.08	4.52	3.98	3.88	4.34	4.44	4.00	4.87
4 Alter density *d*_2_	4.50	5.75	4.31	4.32	5.37	5.39	4.33	5.73
5 Betweenness *b*_ego_	0.895	0.889	0.910	0.900	0.894	0.890	0.899	0.888
6 Effective size *N*_eff_	48.9	49.6	45.1	50.9	45.9	44.9	49.0	40.9
7 Module ratio *M*_ratio_	0.449	0.460	0.467	0.451	0.500	0.511	0.429	0.488
8 Fragmentation index *F*	0.569	0.560	0.608	0.577	0.570	0.635	0.569	0.561
9 Layer diversity of habitat links *H*_h_	0.664	0.659	0.643	0.661	0.666	0.666	0.663	0.662
*IQV* of 9	0.996	0.988	0.965	0.992	0.999	1.000	0.994	0.993
10 Layer diversity of interaction type links *H*_i_	0.409	0.447	0.488	0.422	0.416	0.385	0.415	0.412
*IQV* of 10	0.545	0.595	0.650	0.563	0.555	0.513	0.554	0.550

### 3.2 The ego net through 300 years of change and a future with or without mice

**FIGURE 4:**
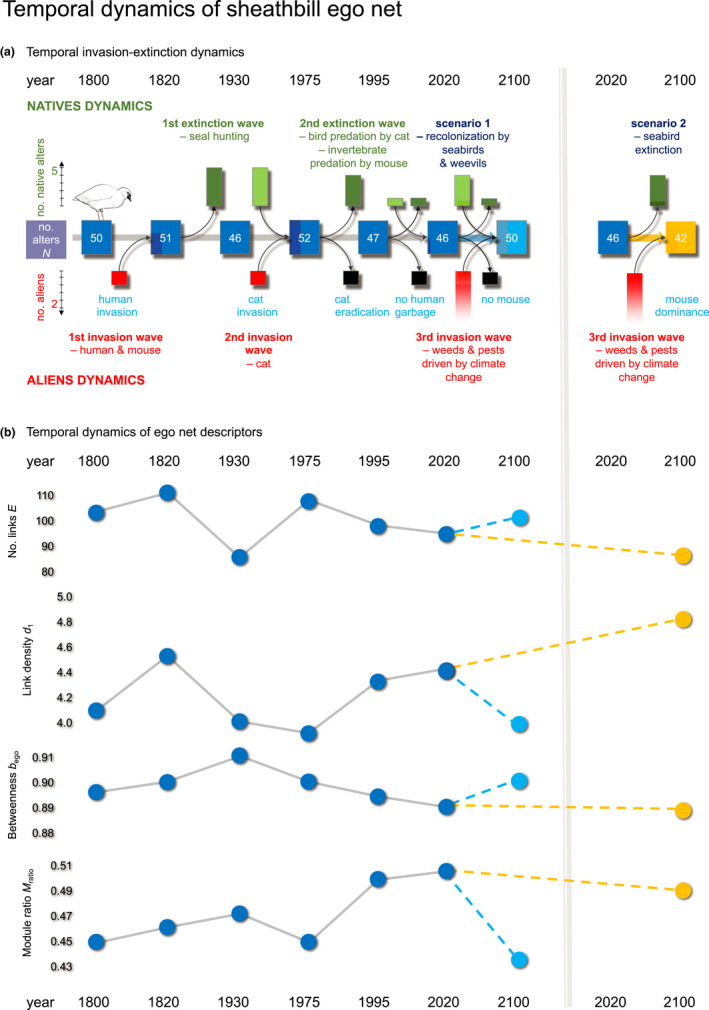
300‐years of temporal dynamics of sheathbill (*Chionis minor*) ego net. Two future scenarios—with and without mouse—are shown. (a) extinction and invasion turnover: Light‐green and dark‐green bars indicate native species, entering or leaving the network since the previous time layer. Red and black bars indicate exotic species, entering or leaving the network since the previous time layer. Major ecological events on Marion are indicated. (b) temporal dynamics of four ego net descriptors

Since the first humans arrived to Marion in 1803, the island has suffered from two major ecological disasters: seal exploitation and cat presence. These two hundred years of ecological history may be visualized and analyzed by the ego net dynamics of some of the key players on the island, such as sheathbill (Figure 4a). I did that by calculating the values of the ten descriptors of the sheathbill ego net for each time slice (Table 1). I expected the descriptors to be partly correlated. This was tested by Person's product‐moment correlation analyses (Figure S1). However, only four of the 45 descriptor pair combinations were significantly correlated (Pearson's/Spearman's; *p* < .05). Thus, almost all descriptors were sensitive to various network aspects of the sheathbill's ecological history, but they did fall into four groups, showing some intra‐group covariation (*p* < .4): Group 1: number of alters *N*, number of links *E*, and effective network size *N*_eff_. Group 2: link diversity among habitats *H*_h_, total link density *d*_1_, and alter–alter link diversity *d*_2_. Group 3: module ratio *M*_ratio_. Group 4: ego betweenness *b*_ego_, fragmentation index *F*‐*index*, and link diversity among interaction types *H*_i_. This group pattern was stronger for a future mouse‐presence scenario than for one without the mouse (Figure S1), that is, descriptors stayed more correlated (synchronized), if the mouse remained on the island. The temporality of one descriptor from each group is shown in Figure 4b.

Network size (*N*, *E*, and *N*eff) followed the invasion and extinction waves (Figure 4a) and showed a grim change in a future, warmer and drier, mouse‐dominated Marion (Table 1).

*M*_ratio_ had the opposite behavior of network size *N*, partially because *N* is included in the denominator in the formula for *M*_ratio_. Each time a human disturbance was removed, modularity recovered. In the future, *M*_ratio_ will stay high, if the mouse stays, but drop if it gets eliminated. This is, at least partially, driven by changes in *N*.

*d*_1_ and *d*_2_ increased with the first wave of invaders, but then decreased continuously until after cat eradication, when their values again rose. In the decades during and after seal hunting, *H*_h_ dropped, but has since remained stable. When seals were removed, marine species in the network became less common, and consequently *H*_h_ got lower. Eliminating the generalist mouse will lower density values.

After 1930, *b*_sheathbill_ decreased, that is, after the return of the seals, and it will continue to do so, if the mouse stays, that is, the sheathbill will lose control. If the mouse gets eradicated, *b*_sheathbill_ may bounce back toward former higher levels.

The temporal dynamics of the descriptors tell in detail, how invasion and extinction affect an ego net (Figure 4) and how descriptors take different trajectories in a mouse‐infested and a mouse‐free Marion (Figure 4b).

## 4 DISCUSSION

### 4.1 Past and presence of the sheathbill and its ego net

I introduced ego net analysis as a tool in ecology and illustrated its applicability by an example. So, to summarize, did my work give any insight about Lesser Sheathbill, which I could not otherwise have achieved? I believe it has—obviously, and below I elaborate on this brief answer.

The ego net of sheathbill is the largest of its kind on Marion (measured as *N* and *E*, *unpublished*), which means, that the sheathbill is a food‐n’‐habitat generalist with a strong ecological integration in its environment. It consumes 46 of its alters and is a resource of four, *viz*. three lice and the Kelp Gull (*Larus dominicanus*), which predates upon sheathbill chicks (Burger, 1980). This high ratio (46:4) indicates, that sheathbill in a variety of ways benefits from its large network (Perry et al., 2018). Since sheathbill is a sedentary bird on the island, more alters, *ceteris paribus*, also add more stability to its resource supply by reducing number and size of windows of scarcity. However, this high level of generalization probably also constrains the bird, because it requires more behavioral plasticity (Burger, 1982, 1984). Using a social network analogy, sheathbill may “suffer” from an overload of environmental information. Altogether, it adds the first brush strokes to a picture of a super generalist, surviving on a small island with only scant resources to offer its inhabitants. Thus, sheathbill becomes a prototype of land birds also found on other small oceanic islands (*e*.*g*., Blanco et al., 2014; 2015; Olesen et al., 2018; Traveset et al., 2015).

The ego net of sheathbill has many alters, lowering link density (*d*_1_ and *d*_2_), which translates into a lower network coherence, that is, slower spread of perturbations, and thus also decreased alter synchrony—meaning an increased uncoupling of alter population dynamics. Weaker synchrony may result in more frequent, but also weaker oscillations in levels of the various resources, again stabilizing the palette of food—there will always be something to eat somewhere to a nonpicky bird so to speak. Generally, lower link density means less information redundancy (network effective size *N*_eff_ is high, *i*.*e*., *N* ~ *N*_eff_), less “ecological backup,” and more novelty. Besides a high number of alters, low link density is also a consequence of many forbidden interactions (Izquierdo‐Palma et al., 2021), which, however, may be selected against in resource‐poor environments, such as on islands, for example *f*, by reducing mismatches in phenology among interacting partners (longer diurnal and seasonal activity period), habitat (more habitat border crossing), and diet (more omnivory, opportunism).

Whereas presence of forbidden links is background for a discussion of match/mismatch among nodes; the issue of structural holes is related to a discussion of ego control. A prominence of structural holes tells, that ego is in control, being a broker between groups (“a divide‐n’‐conquer strategy”) (Burt, 1992). A high betweenness value tells the same. Most “information” has to pass ego, that is, ego's alter control is high, whereas alter–alter control is low. Through its high betweenness, ego acts as conduit for indirect effects among alters (Granovetter, 1973; Valente & Fujimoto, 2010). Thus, effective size of an ego net and its betweenness are also estimates of the importance of indirect interactions among alters, mediated by ego. However, the ecological advantages and disadvantages of forbidden links and structural holes need more study, especially in a climate change context.

If an ego has strong links to two alters, the likelihood increases that these also get connected, at least with a weak link (Granovetter, 1973), that is, a structural hole gets closed (“a triadic closure”) (Burt, 1992). Sheathbill is, for example, a predator of the two decomposers Kerguelen Earthworm and Pringlea Moth and consequently, if population size of these two increases and thus their level of detritivory, they may face a weak apparent competition and/or mutualism (Holt & Bonsall, 2017). This is perhaps a topological subtlety, but other triadic closures may initiate serious repercussions throughout the network. Since arrival of humans to Marion, the mouse has been predating on invertebrates, that decompose seabird‐derived detritus. These invertebrates are linked to both mouse and seabird detritus. In 2003, the mouse made a triadic closure, by establishing a new and absolutely revolting direct link by its predation of seabird chicks (Dilley, 2018). During the coming years, this may push the sheathbill ego net into an alternative regime with dire consequences to the complete island network.

Like any real network, the sheathbill ego net is modular (*N*_m_, *M*_ratio_, and modularity level *Q*) and probably often strongly fragmented (*i*.*e*. high fragmentation index). Drivers of resolution of modular structure are network dimensions, for example, by adding data about habitat and interaction type, *Q* increased from 0.34 to 0.79 (*unpublished*). Together these two dimensions sorted the assembly of alters into seven modules. The predation module in all three habitats even had a deeper, hierarchical structure, which might be driven by other, unknown dimensions. This observation suggests further research lines (Olesen et al., 2010).

My earlier discussion of asynchrony has to be modified by information about modular heterogeneity. We do not just talk about level of synchrony in the network; the modules of the network also vary in synchrony, that is, synchrony might be high inside one module, but low in another. Each of the seven modules in Figure 3b occupy a module niche, for example, the land detritivory module, consisting of 13 alters and ego. This module is running on detritus—or actually two kinds of detritus, *viz*. seabird guano and leftovers and swags from penguin colonies. The next step will be to gather information about the natural history of the species in each module, in particular their trait diversity. Finally, we may proceed and make deductions from traits to the overall functionality of a module, which is the outcome of the combined ecology of its species, that is, toward emergent or collective properties (*e*.*g*., see Anderson, 1972; the title of this paper being very telling—“More is different”). Such properties are well researched in physics, genetics, and molecular biology and seen, at least from a distance, these properties show cross‐disciplinary similarities. The seven modules of sheathbill's ego net are integrated into each other by connector species, that operate across several layers within a dimension (Olesen et al., 2018), for example, seabirds crossing back and forth between sea and land. In conclusion, ecological module thinking is now moving from description of modularity (level of modularity, number of modules, and node roles) toward an understanding of structural–dynamical relationships among modules and their functional characterization in space and time.

Dimensions and their layers have implications for metapopulation and ‐community dynamics. In the sheathbill ego net, as everywhere else in nature, populations fluctuate in size, and I imagine such fluctuations drive a kind of “jellyfish‐like” pulsation of the sheathbill net in the 3‐D space: habitat–interaction type–time. Changes in size of the population of sheathbill cause expansion and retraction of the network along its dimensions. Ego and alters may show source‐sink dynamics between adjacent layers and dimensions, for example, an increasing sheathbill population may force inferior individuals from land (the source) out into the tidal zone (a sink) or from less predation to more herbivory. In addition, moving to another layer may have a rescue effect to a species, assuming that its antagonists are delayed in their response. This dynamics trickers pulses throughout the network, for example, when Patagonian tooth‐fish fishery in the Southern Ocean decimates penguins (Nel et al., 2002, 2003), less detritus is produced on Marion with a dimension‐layer reshuffling among invertebrate decomposers, being the essential food to sheathbill (Chown et al., 2002).

Based on my application of ego net analysis, I conclude, that a new and deeper understanding of the ecology of the focal bird was achieved, especially of the structure and dynamics of its biotic context, that is, level of control of resource synchrony, interactions, modularity, and dependence upon niche dimensions.

### 4.2 Future of the sheathbill and its ego net

As ecologists, we aim at understanding present‐day ecology of key species, but in a fast‐changing world, we increasingly need to predict how such species will steer into the future (Krebs, 2009). So, how does the sheathbill's future look like? Very dark—perhaps. It all depends on mice, climate, and new invaders.

The long research tradition on Marion with its rich scientific output made it feasible to track sheathbill's ego net through the last 200 years, but also further out to the end of this century. (This history is detailed below in Appendix 2). First, sealers vehemently killed the seals, and mice thrived on a broad and mixed diet of detritus‐feeding invertebrates and plant material, but apparently without interfering with sheathbill. (Details of the temporal dynamics of the mouse ego net are quantified below in Table A1 in Appendix 1). Second, the cat was introduced in 1949 by scientists and during the next four decades it caused a true bloodshed among seabirds. After 1991, the cat was out and the mouse population increased, facilitated by longer and warmer seasons (Chown & Smith, 1993; Frenot et al., 1997; McClelland et al., 2018; Smith & Steenkamp, 1990). On a future, increasingly mouse‐infested Marion, the sheathbill network will shrink in size, get denser, and more fragmented, and ultimately—the analysis predicts—sheathbill will lose its alter control. The mouse, on the other hand, is seemingly doing well, because of warmer climate and its new food—seabird toddlers (Dilley, 2018). Consensus among conservationists is that to functionally restore the island's ecological network, the first but also most crucial step must be to exterminate the mouse, and plans (The Mouse‐free Marion Project *Saving Marion Island's Seabirds*) to do so should be realized in the austral winter of 2024, if an ongoing crowd‐funding initiative becomes successful (https://mousefreemarion.org; Parkes, 2014).

If the mouse gets eradicated, descriptors predict the sheathbill ego net will attain its prehuman topology (Jones & Ryan, 2010). However, this is far from certain—details about system reversibility remains unclear, because alters are much more than their number—some natives are going to be replaced by invaders with a different natural history background—robust globetrotters replacing cold‐ and humidity‐adapted natives. Future invaders could be entirely new taxonomic groups, for example, ants might become a serious threat (*e*.*g*., Davis et al., 2008). Their impact upon Marion birds and especially the native, flightless invertebrate fauna may turn out to be a final apocalypse. Other invaders bring new interaction types, for example, pollination (Convey et al., 2010). A lingering question will be—are we really going to see a reversible shift to a pre‐1800 topology or will it be a shift to yet another alternative regime (Mulder et al., 2009; Scheffer et al., 2001)?

On a mouse‐free Marion, the sky may look bright but not the future. Air and sea surface temperatures already commenced a rise in the late 1960s by an average of 0.04°C per year (Smith & Steenkamp, 1990), and about the same time, precipitation began—from a level of 3000 mm annually—to drop with a mean annual rate of 25 mm (Smith et al., 2002; Summer et al., 2004). Actual figures vary among references and the fashion of monitoring has changed. However, all agree, that Marion gets warmer and drier and throughout the present century this trend continues (Meehl et al., 2007). A warmer climate will redirect sea currents and marine life, affecting foraging of seabirds. The Marion seabird fauna may shrink, causing collapses in detritus accumulation and invertebrate decomposer fauna. Exact magnitude and direction of this regime shift are unclear. The expected historical and future dramatic, nonlinear dynamics of the Marion network is also a well‐known phenomenon elsewhere in the world, and it makes the task of forecasting any population trends quite futile (Clarke & Luis, 2020), because, unfortunately, in complex systems the past hardly tells us much about the future.

## 5 CONCLUSIONS

An ecological ego net approach draws a detailed map of information about a key ego player and its interactions in an ecosystem, making it feasible, for example, to track disruptive consequences of disturbances such as invasions and extinctions, and to pinpoint potential places of conservation action. Therefore, an ego net is a map from which new ecological insight can be read and more detailed questions formulated.

The ego net analysis documents quantitatively how sheathbill plays a key role on Marion with its high topological generality and plasticity, that is, omnivory, opportunism, and wide habitat use, and with a low direct contact to invaders (Burger, 1980; Huyser et al., 2000; Jouventin et al., 1996; McClelland, 2013; Ramos‐Jiliberto et al., 2012; Verheyden & Jouventin, 1991). Its continued presence is indeed the best evidence of its robustness. However, if the mouse remains on the island, the sheathbill is expected to be doomed, mainly because the mouse now begins to consume its resource base, guano‐producing seabirds. Thus, the sheathbill ego net has up to the turn of this century been resilient to the waves of extinction outside its own periphery, but is now becoming vulnerable. In general, sensitivity of system hubs like sheathbill against attack is a defining characteristic of the break‐down of scale‐free networks (Barabási & Oltvai, 2004).

A successful restoration will be hampered by extensive hysteresis, that is, recovery time (Scheffer et al., 2001), not only because of new invaders, but also because of ongoing warming, which will draw a different resource map of the surrounding sea (Chown & Smith, 1993; Frenot et al., 1997; Smith & Steenkamp, 1990). These large‐scale changes are predicted by all 2100 climate scenarios (Meehl et al., 2007), and how this is going to affect tiny Marion and other ecologically similar islands in the Southern Ocean, we do not know.

The present story of a small bird on a small island may seem esoteric and of marginal interest, but its action takes place in the Southern Ocean with its Antarctic Circumpolar Current—the largest of its kind in the world. The current is called “the world's climate engine” and as stated clearly by the oceanographer J. L. Russell (cited in Fountain & White, 2021): “From no perspective is there any place more important than the Southern Ocean. There is nothing like it on Planet Earth.” Therefore, its small islands, for example, Marion, can be seen as climate sensors of global relevance. This study shows the organization and fragility of natural complexity on such a small, supposedly simple island at high latitude and far from any continent, that is, the complexity of simplicity; and here, in a part of the world under fast transition, a poor flyer and thus physically “trapped” species, the sheathbill, is stuck in between a pair of opposing processes—the risk of extinction as its food items one by one dwindle, whereas its enemy the mouse plays.

## AUTHOR CONTRIBUTIONS

**Jens Mogens Olesen:** Conceptualization (equal); Data curation (equal); Formal analysis (equal); Funding acquisition (equal); Investigation (equal); Methodology (equal); Project administration (equal).

## CONFLICT OF INTEREST

None.

## Supporting information

Fig S1

Supplementary Material

## Figures and Tables

**FIGURE A1:**
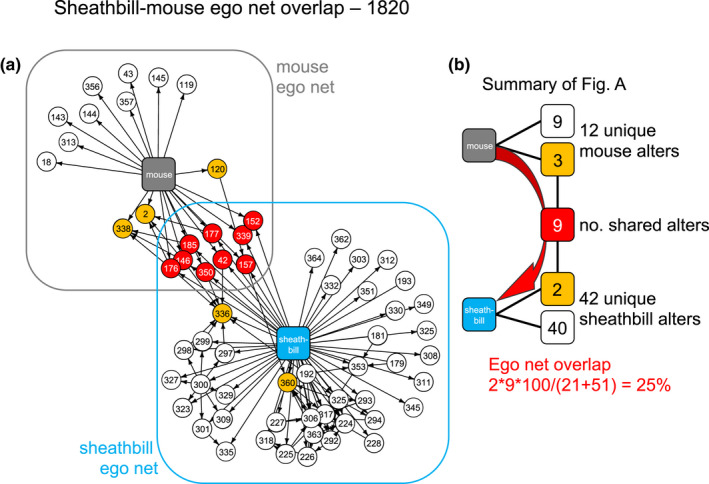
(a) year 1820 ego nets of House Mouse (*Mus musculus*) and Lesser Sheathbill (*Chionis minor*) and their alter overlap (red taxon nodes). Yellow nodes are mouse and sheathbill alters, that are linked to shared alters. (b) Summary of *A*. The ego net overlap = 100 · 2 · no. shared alters/(no. sheathbill alters + no. mouse alters). (See Supplementary Material S1 for scientific names)

**FIGURE A2:**
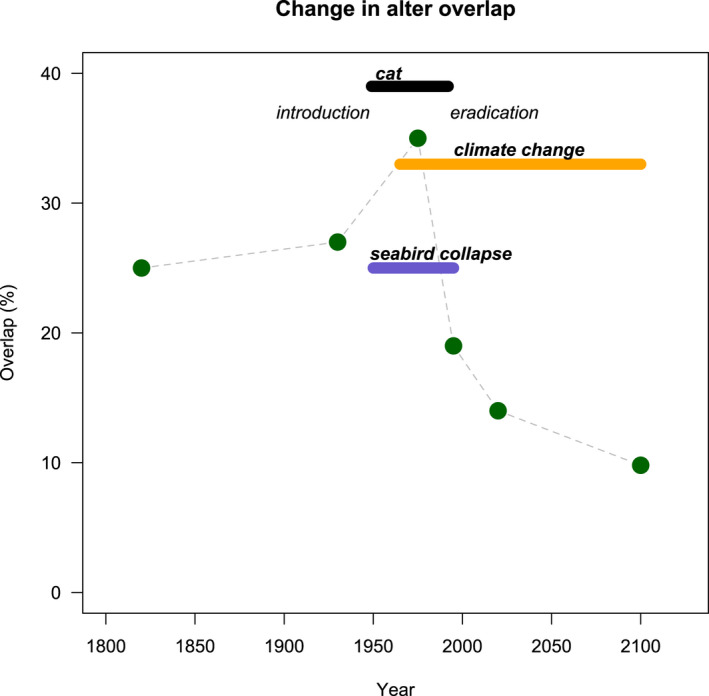
Temporal dynamics of the mouse‐sheathbill ego net overlap (*Mus musculus*–*Chionis minor*). Major ecological events on Marion are indicated (for details about how to calculate overlap, see Figure A1)

## Data Availability

All data are provided in the Supplementary Materials S1–S5.

## APPENDIX 1

### EGO NET ANALYSIS OF HOUSE MOUSE

#### The House Mouse ego net through 300 years

The 300‐year temporal dynamics of descriptors of the ego net of the House Mouse (*Mus musculus*) is given in Table A1. There are striking differences between the dynamics of the Lesser Sheathbill (*Chionis minor*) and mouse ego nets, for example, the mouse modularity ratio *M*_ratio_ is twice as high. Thus, although the mouse has fewer alters, it has almost a similar number of modules as the sheathbill, which means it operates in several independent trophic niches and has a high control over its food resources. The very high betweenness values support this conclusion. The high *M*_ratio_ is perhaps an important indicator of sustained invasion success.

**TABLE A1:** 300‐years of dynamics of ego net descriptors for House Mouse. The year–2100 scenario is with a presence of mouse (unpublished material)

Descriptors	1820	1930	1975	1995	Today	2100 +mouse
1 Number of alters *N*	18	18	21	15	20	15
2 Number of links *E*	25	25	29	20	27	20
3 Total density *d*_1_	7.31	7.31	6.28	8.33	6.43	8.33
4 Alter density *d*_2_	6.80	6.80	4.97	5.88	4.35	5.88
5 Betweenness *b*_ego_	0.931	0.931	0.938	0.937	0.955	0.937
6 Effective size *N*_eff_	17.6	17.6	20.6	14.7	19.7	14.7
7 Module ratio *M*_ratio_	0.647	0.647	0.700	0.643	0.631	0.600
8 Fragmentation index *F*	0.105	0.105	0.095	0.076	0.042	0.067
9 Layer diversity of habitat links *H*_h_	0.077	0.077	0.067	0.180	0.263	0.180
*IQV* of 9	0.115	0.115	0.100	0.270	0.395	0.270
10 Layer diversity of interaction type links *H*_i_	0.461	0.461	0.452	0.445	0.359	0.445
*IQV* of 10	0.614	0.614	0.602	0.593	0.479	0.593

#### Indirect interactions between Lesser Sheathbill and House Mouse

An indirect interaction between a pair of species is mediated by a third species with whom they both interact, that is, according to my definition two species interact indirectly, if they are two link steps apart. For example, when a predator interacts directly with herbivores and detritivores, it may interact indirectly with other predators and with plant and detritus. As omnivores, both mouse and sheathbill do so. Up to now, sheathbill and mouse have not interacted directly, but they have done so indirectly through their sharing of alters (Figure A1). During the 200 years of observation, overlap of the two ego nets peaked during the cat dynasty (Figure A2). Later, the intense predation of native invertebrate populations by the mouse and the reduction of seabird detritus accumulation have lowered the ego net overlap, and an expected future increase in mouse predation of seabirds will enforce this development. This is explained by the disappearing invertebrate food shared by the two. Thus, the mouse affects sheathbill negatively by pushing it out of their alter overlap.

Table A2 gives the potential extent of indirect interactions between sheathbill or mouse and the entire Marion Island network, which has a recorded 368 nodes and 767 trophic links (*unpublished data*).

**TABLE A2:** Direct (1 link away) and indirect interactions (2 links away) between House Mouse or Lesser Sheathbill and the total Marion Island network

	Total number
Marion Island nodes	368
Marion Island links	767

	No. direct links	No. indirect links	Total no. links
Sheathbill	54	156	210
Mouse	30	80	110
Sheathbill (pct. of 767)	7.0		
Mouse (pct. of 767)	3.9		

## APPENDIX 2

### HISTORICAL ANALYSIS

I use node and interaction data to construct 300 years of ecological history of the ego net of the Lesser Sheathbill (*Chionis minor*), *viz*. from before whalers’ arrival in the summer of 1803/1804 to a projected 2100 (Supplementary Material S1). In order to do so, I analyze a temporal sequence of eight networks: prehuman 1800, *c*. 1820, *c*. 1930, *c*. 1975, *c*. 1995, 2020 (today), and two 2100 scenarios (with and without mouse). The actual years are only labels for periods delimiting major biotic events, such as an invasion or eradication of an important pest species. Each time‐slice network pools the three habitats—land, tidal zone, and sea, and all interaction types.

#### Description of periods

**I: Prehuman period as an ecological baseline (−1800, time‐slice label “1800”)** ended in the summer of 1803/1804, when the first seal hunters and whalers arrived (Cooper, 2008, 2008). Prehuman Marion had vast colonies of seabirds and sea mammals, which produced large amounts of organic matter at their colony and wallow sites. Thus, this time‐slice has no human‐mediated introductions.

**II: Period of commercial seal and penguin exploitation (1803‐*c*. 1860, time‐slice label “1820”)** began with the arrival of the first sealers and whalers in the summer of 1803/1804. Their hunting of seals (*Arctocephalus* spp., *Mirounga leonina*) and birds (especially of King Penguin, *Aptenodytes patagonicus*) was very dramatic and went on until seals were driven to “economical extinction” in the 1860's (Cooper & Headland, 1991), but continued more sporadically until the 1930's (Kearley, 1987). The effects of this on the seal populations and the entire ecosystem of the island lingered on into the 20th century.

Besides humans, the most notorious earlier invader was the House Mouse (*Mus musculus*), which probably arrived shortly after 1818 (Watkins & Cooper, 1986). The mouse reduced the abundance of the sheathbill's prey, the macroinvertebrate decomposers (Smith et al., 2002, 2002), and also interacted directly with plants as herbivore and granivore (Chown & Smith, 1993), but apparently never directly with the sheathbill.

For this period, I assume that plant and animal invaders with early or unknown arrival time got to the island.

**III: Period without seals (*c*. 1860–1948, time‐slice label “1930”)** began after “the economical extinction” of seals (Cooper & Headland, 1991), covered a slow return of seals, and ended with the building of a weather station in 1948 and the introduction of the cat in 1948/1949. The data set is similar to that of period II, but seals and King Penguin and their interactions are removed, while a second wave of alien plants and invertebrates is included.

**IV: Period of cat predation (1949–1991, time‐slice label “1975”)** began with the construction of a weather station and the disastrous cat introduction in the summer of 1948/1949, and ended with the eradication of the cat in 1991.

During this period, the Marion climate got warmer and drier, and the surrounding sea also got warmer (Mélice et al., 2003; le Roux & McGeoch, 2008). The summer snowline, which was at 600–850 m above sea level in 1954–1961, went up to 950 m in 1971, and today, it is “above” the altitude of the island. From 1950 to 2010, global seabird populations declined 70%, a development also noted on Marion (Paleczny et al., 2015). Simultaneously, Marion experienced a steady arrival of new alien plants and invertebrates, increased scientific exploration, and more deposition of human garbage. The latter was exploited by sheathbill and other birds. Around 1975, it was estimated that a population of 2000 cats yearly killed 4,50,000 seabirds (van Aarde, 1980), an almost incomprehensible number. In addition, albatrosses were affected by long‐lines fishery in the 1970's and 1980's (Nel et al., 2002, 2003). The declining seabird populations meant less soil erosion and marine input, harming invertebrate decomposers. One light in the darkness was an increase in fur seals (Hofmeyr et al., 1997). However, the overall result was a degradation of the sea‐land bird connection or, as it has been termed, “the life support system” of the island (Pakhomov et al., 2009). For this period, all remaining invaders and the seals and their interactions are included. I keep the macroinvertebrates in the dataset although their abundances declined. With the available data, it will always be somewhat arbitrary when to include or exclude invertebrates. I removed Pringlea Moth (*Pringleophaga marioni*) and weevils (Curculionidae) from the database, when relevant references gave me the impression that their ecological importance had disappeared, but my decisions are certainly open to critique.

**V: Period after cat eradication (1991‐*c*. 2001, time‐slice label “1995”)** covered the first decade after cat eradication in 1991 and ended with the first observations of bird predation by mice in 2003 (Dilley et al., 2016; Jones & Ryan, 1990, 2010)—“when the cat's away, the mouse will play,” and it certainly did so.

Both increasing number of mice due to cat eradication and warming meant fewer native invertebrates and thus less winter food to sheathbill (Huyser et al., 2019, 2000). In addition, cat predation on burrowing petrels lowered transfer of detritus to below‐ground decomposing invertebrates, which also harmed the sheathbill. Finally, fewer penguins, which supply sheathbills with food, was also malevolent. In spite of these problems, sheathbills still maintained a population of ~1400 breeding pairs in this period and the next one (Burger & Kirwan, 2020).

The cat, a few seabirds, native grasses and Cyperaceae, and their interactions are removed from the dataset (Gremmen et al., 1998). The impact of mouse predation on seabirds is not yet included. The macroinvertebrates stay in the dataset, except for Pringlea Moth and weevils (Chown et al., 2002), although their general abundance is assumed to be low, because of mouse predation and reduced detritus deposition by birds.

**VI: Period with mouse predation of birds (*c*. 2001‐today, time‐slice label “2020”)** began in 2001 and ended “today.” From 1979 to 2011, the lowland mouse population increased 5–6 times (McClelland et al., 2013, 2018) due to climate change, specifically earlier seasons and more dry days. During the same period, its invertebrate food dropped more than two orders in abundance, especially adult weevils got rare in the diet (McClelland et al., 2013, 2018). In some habitats, these animals disappeared almost completely, whereas in other habitats their abundance is still 1/3 of their abundance back in 1976.

In 2003, the mouse was observed to consume eggs and attack juveniles of seabirds, especially during winter; a change simultaneously seen on other sub‐Antarctic islands (Angel et al., 2009; Dilley et al., 2016; Jones & Ryan, 1990, 2010). In 2015, *c*. 5% of summer‐breeding albatross fledglings were killed by mice.

The south‐Antarctic Front moves southward, benefitting offshore‐foraging seabirds, but harms most inshore birds. As fish‐eaters, penguins interact with fishery, resulting in bycatch and food competition. This causes repercussions inland, because penguins are dominant producers of detritus. Seals are now assumed to have reached prehistoric population sizes. Several seabirds, native grasses and Cyperaceae, *Acaena magellanica*, and their interactions are removed (Gremmen et al., 1998). Predation by mice on seabirds is included. The macroinvertebrates stay in the dataset, except for Pringlea Moth and weevils. The general abundance of macroinvertebrates is, however, assumed to be low and local extinction might take place.

**VII(no** **mouse, but all other invaders included): Future climate change without mouse (today‐2100, time‐slice label “2100, no mouse”)**

In the austral winter of 2024, there are plans to initiate an eradication program of the mouse by aerial baiting (see 4.2). Thus, in this time slice, I remove the mouse. Southern Rockhopper Penguin (*Eudyptes chrysocome*) is also excluded (Pakhomov & Chown, 2003). However, I let invertebrates, and native grasses and Cyperaceae recover. Survival of weevils may be low, because of higher temperatures and lower precipitation (Klok & Chown, 1997; van der Merwe et al., 1997).

**VII(mouse** **and all other invaders included): Future climate change with mouse (today‐2100, time‐slice label “2100, mouse”)**

In this alternative time slice, I keep the mouse and remove all birds, except the Brown Skua (*Catharacta antartica*), sheathbill, and three of the penguins (Crafford et al., 2003, 2009). The macroinvertebrates stay in the dataset, except Pringlea Moth and weevils, although the general abundance of macroinvertebrates is assumed to be low, because of more enemies (mice), less food (seabird detritus), and a more unfavorable climate (van der Merwe et al., 1997). Native grasses and Cyperaceae, *Acaena magellanica*, and their interactions are removed (Gremmen et al., 1998).
