# Supplementary material for: Ego network analysis of the trophic structure of an island land bird through 300 years of climate change and invaders
Source: Ecol Evol. 2022 May 20;12(5):e8916. doi: 10.1002/ece3.8916 (PMC9121045; doi:10.1002/ece3.8916)
Supplement: Supplementary file 1 — Fig S1 [file ECE3-12-e8916-s001.pdf]

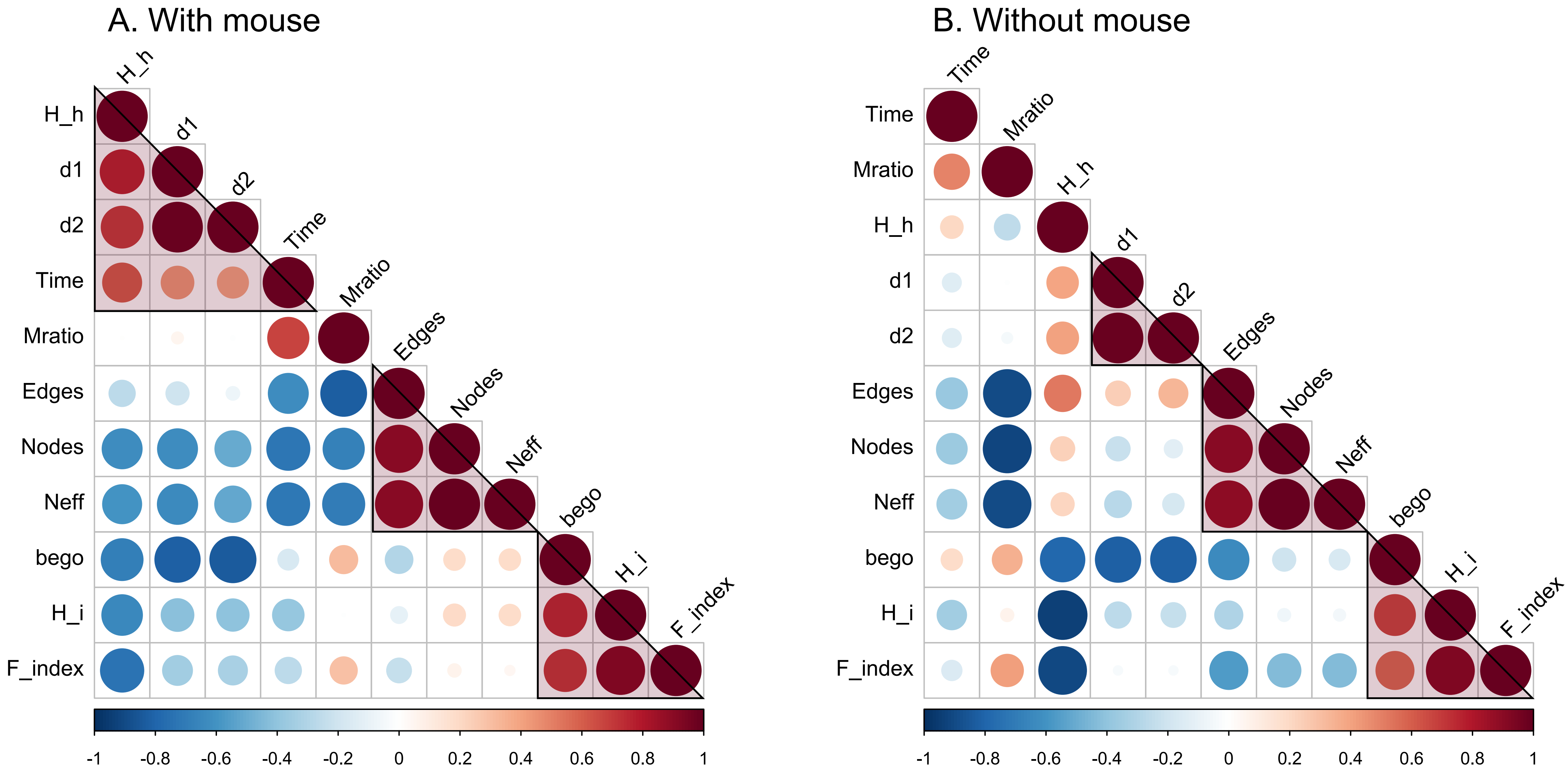

Supplementary Figure 2. Pearson's product-moment correlograms with (left) and without (right) mouse in year 2100. Nodes, no. alters *N;* Edges, no. edges *E*; d1, total density; d2, alter density; bego, betweeness *b* of ego; Neff, effective size; Mratio, module ratio M_{ratio}; F–index, fragmentation index; H–h, layer diversity of habitat links; H–i, layer diversity of interaction type links. Intensity of the colours is proportional to the correlation coefficients. Colour legend below each correlogram shows the correlation coefficient and its corresponding colour.
